# PACAP and VIP Modulate LPS-Induced Microglial Activation and Trigger Distinct Phenotypic Changes in Murine BV2 Microglial Cells

**DOI:** 10.3390/ijms222010947

**Published:** 2021-10-11

**Authors:** Jocelyn Karunia, Aram Niaz, Mawj Mandwie, Sarah Thomas Broome, Kevin A. Keay, James A. Waschek, Ghaith Al-Badri, Alessandro Castorina

**Affiliations:** 1 Laboratory of Cellular and Molecular Neuroscience (LCMN), School of Life Sciences, Faculty of Science, University of Technology Sydney, Sydney, NSW 2007, Australia; jocelyn.karunia@uts.edu.au (J.K.); aram.niaz@health.nsw.gov.au (A.N.); mmandwie@cmri.org.au (M.M.); sarah.j.thomasbroome@student.uts.edu.au (S.T.B.); g.al-badri@unsw.edu.au (G.A.-B.); 2 School of Medical Science, [Neuroscience] and Brain and Mind Centre, The University of Sydney, Sydney, NSW 2006, Australia; kevin.keay@sydney.edu.au; 3 Intellectual Development and Disabilities Research Centre, Semel Institute for Neuroscience and Human Behaviour/Neuropsychiatric Institute, David Geffen School of Medicine, University of California-Los Angeles, Los Angeles, CA 90095, USA; jwaschek@mednet.ucla.edu

**Keywords:** microglial activation, PACAP, VIP, morphological analyses, BV2, inflammatory markers, GFAP, Iba1

## Abstract

Pituitary adenylate cyclase-activating polypeptide (PACAP) and vasoactive intestinal peptide (VIP) are two structurally related immunosuppressive peptides. However, the underlying mechanisms through which these peptides regulate microglial activity are not fully understood. Using lipopolysaccharide (LPS) to induce an inflammatory challenge, we tested whether PACAP or VIP differentially affected microglial activation, morphology and cell migration. We found that both peptides attenuated LPS-induced expression of the microglial activation markers *Iba1* and *iNOS* (### *p* < 0.001), as well as the pro-inflammatory mediators *IL-1β*, *IL-6*, *Itgam* and *CD68* (### *p* < 0.001). In contrast, treatment with PACAP or VIP exerted distinct effects on microglial morphology and migration. PACAP reversed LPS-induced soma enlargement and increased the percentage of small-sized, rounded cells (54.09% vs. 12.05% in LPS-treated cells), whereas VIP promoted a phenotypic shift towards cell subpopulations with mid-sized, spindle-shaped somata (48.41% vs. 31.36% in LPS-treated cells). Additionally, PACAP was more efficient than VIP in restoring LPS-induced impairment of cell migration and the expression of urokinase plasminogen activator (uPA) in BV2 cells compared with VIP. These results suggest that whilst both PACAP and VIP exert similar immunosuppressive effects in activated BV2 microglia, each peptide triggers distinctive shifts towards phenotypes of differing morphologies and with differing migration capacities.

## 1. Introduction

Microglia are central nervous system (CNS) resident, macrophage-like cells whose activities are highly relevant in neuroimmune interactions, especially after immune insult or trauma to the brain [1,2]. In particular, these cells play important roles in modulating both the innate and adaptive immune responses through their ability to produce and srete several inflammatory mediators following polarization, via various Toll-like receptors (TLRs), (i.e., TLRs 1–9) [3,4]. In the CNS, the process of neurodegeneration is characterised by morphological, metabolic and functional neuronal changes that result in premature, progressive and chronic neural cell loss [5,6,7,8]. It has been recently recognized that uncontrolled and long-lasting neuroinflammation might be responsible for the progressive CNS degeneration seen in many neurological conditions [9,10]. A compelling hypothesis is that microglial cells play a particularly important role in the aetiology and/or progression of neurodegenerative and autoimmune diseases [2,11,12,13,14,15,16,17,18]. Within the healthy CNS, microglia are said to exist in a “resting” state. In this state, their morphology in tissue appears as a cell with a small soma and ramified processes. In fact, the smaller processes’ “filopodia” are motile and appear to survey the CNS parenchyma continuously [19]. However, on interacting with invading pathogens or after CNS injury, microglial cells transition to an activated, or polarised, state by undergoing a series of neurochemical and morphological changes, including the enlargement and flattening of the soma and the shortening and thickening of the cellular processes [20]. At the biochemical level, microglial cells can be activated to either a pro-inflammatory M1 state, or an anti-inflammatory M2 state [21]. When cells are in the pro-inflammatory M1 state, they produce and secrete several pro-inflammatory cytokines, including tumor necrosis factor (TNF), interleukin (IL)-1β and IL-6, whereas if cells switch to the M2 state they aid in controlling the inflammatory microenvironment by secreting anti-inflammatory cytokines and growth factors, thereby inhibiting inflammation and promoting repair [1,8,22]. It is noteworthy that the production of cells with the M1 phenotype is a necessary step in CNS repair, regardless of their established associations with neurodegeneration [23]. In fact, M1 polarisation is beneficial in the short-term for the survival of neurons, where microglia are able to actively phagocytose cell debris and recruit immune cells to the inflamed CNS compartment. However, a prompt shift to the M2 phenotype or a return to the resting state is required to prevent excessive and prolonged M1 activation [21,24]. There are a number of proposed mechanisms that contribute to the initial activation of the M1 phenotype, including microglial recognition of damage-associated molecular patterns (DAMPs) or pathogen-associated molecular patterns (PAMPs) [8].

To date, the regulatory mechanisms underlying the control of microglial behaviours during inflammation are not fully understood [25]. Multiple studies have shown that uncontrolled M1 microglial activation leads to detrimental effects in the CNS [14,25,26,27,28]. Unfortunately, there have been no treatments identified that are able to halt or prevent persistent microglial activation, and almost all attempts to do this have been unsuccessful.

The neuropeptides PACAP and VIP have gained more interest within the CNS due to their wide range of neuroprotective functions as well as their widespread CNS localisation [29,30,31,32,33,34,35,36]. PACAP and VIP exert their biological effects through their interactions with three G protein-coupled receptors (GPCRs): PAC1, VPAC1 and VPAC2 [37]. PACAP binds to PAC1 with about 100-fold higher affinity than VIP, and both peptides have equally high affinities for both VPAC1 and VPAC2 [38]. PACAP and VIP receptors are expressed in microglia, astrocytes, brain endothelial cells, macrophages, oligodendrocyte progenitors and Schwann cells, and thus are able to elicit cell-specific biological functions in both the CNS and the periphery [30,31,33,35,39,40,41,42].

In addition to their well-known neuro- and glio-protective functions, at nanomolar concentrations, PACAP and VIP are also able to modulate immunological function [43,44,45,46].

Each peptide has been shown to inhibit pro-inflammatory cytokines and neurotoxic factors secreted by activated microglia in a model of PD and brain injury [47,48]. Additionally, in an in vivo model of brain trauma, VIP inhibited microglial-derived pro-inflammatory cytokines, including IL-1β [39].

While many of the protective activities of PACAP and VIP on neurons and glial cells have been identified [32,36,49], the question of whether there are any differences in the way microglial cells respond to PACAP or VIP to elicit their anti-inflammatory roles has not been fully explored. To address this issue, in the current study, we used BV2 cells, an immortalized, murine microglial cell line sharing several biochemical properties with primary microglia [50]. BV2 microglia is broadly used as a viable cellular model to investigate the anti-inflammatory properties of several compounds [51,52]. BV2 cells were activated in vitro using LPS, and cells were administered with either PACAP or VIP at concentrations previously identified to be effective; this was followed by biochemical, morphological analyses and assessment of cell migration. Our approach allowed us to reveal biological differences in the cellular response to each of these neuropeptides, in addition to demonstrating that each of the peptides possess robust anti-inflammatory properties.

## 2. Results

### 2.1. Dose–Response Effects of LPS on the mRNA Expression of Pro-Inflammatory Cytokines and Microglia Activation Markers in BV2 Cells

**Figure 1:**
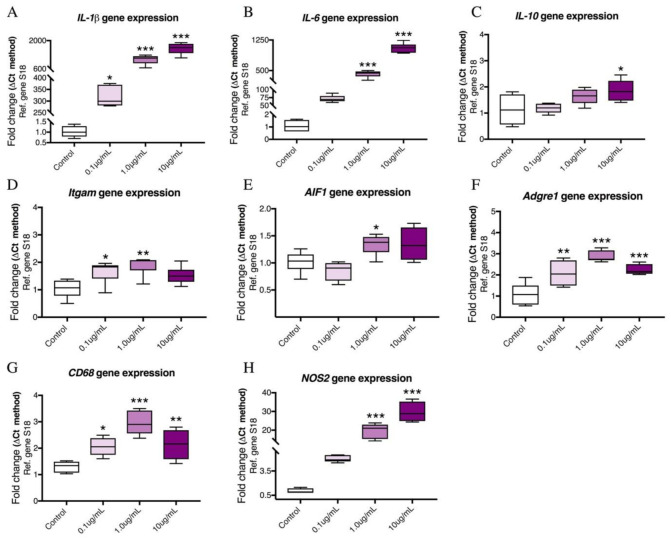
LPS titration experiments showing dose-dependent changes in mRNA expression levels of pro-inflammatory markers in BV2 cells. Murine BV2 cells were serum starved for 24 h prior to treatment with 0.1 μg/mL, 1.0 μg/mL and 10 μg/mL of LPS for 24 h, shown with no LPS controls. RNA was extracted by TRISURE reagent and reverse transcribed into cDNA. The mRNA levels of (**A**) *IL-1β*, (**B**) *IL-6*, (**C**) *IL-10*, (**D**) *Itgam*, (**E**) *AIF1*, (**F**) *Adgre1*, (**G**) *CD68* and (**H**) *NOS2* were determined by real-time qPCR, using S18 as an internal housekeeping gene. Levels were quantified using the Delta-CT method. Data represent means of 4–6 samples for each group. Results are expressed as mean ± SEM. * *p* < 0.05, ** *p* < 0.01, *** *p* < 0.001 vs. control as determined by one-way ANOVA followed by Dunnett’s post hoc test. (*Gene*: protein acronyms): *IL-1β*: Interleukin 1β, *IL-6*: Interleukin 6, *IL-10*: Interleukin 10, *Itgam*: CD11b, *AIF1*: Iba1, *Adgre1*: F4/80, *CD68*: Cluster of differentiation 68 (CD68), *NOS2*: inducible nitric oxide synthase (iNOS), *S18*: ribosomal protein S18.

In order to establish the most suitable concentration of LPS for triggering a robust inflammatory response in the BV2 microglial cell line, we conducted real-time quantitative polymerase chain reaction (qPCR) to measure the gene expression of a panel of pro-inflammatory mediators (*IL-1β*, *IL-6*, *Itgam*, *AIF1*, *Adgre1*, *CD68* and *NOS2*) and the anti-inflammatory gene *IL-10* at increasing concentrations of LPS (0.1, 1 and 10 μg/mL). When LPS was used at a concentration of 0.1 μg/mL, there was only a significant increase in *IL-1β* (F (3,17) = 78.95, * *p* < 0.05), *Itgam* (F (3,19) = 5.373, * *p* < 0.05), *Adgre1* (F (3,17) = 18.54, ** *p* < 0.01) and *CD68* (F (3,20) = 16.54, * *p* < 0.05) (Figure 1A,D,F,G), while the remaining genes examined, *IL-6*, *IL-10*, *AIF1* and *NOS2*, were unaffected (*p* > 0.05, Figure 1B,C,E,H). At 1.0 μg/mL, LPS significantly increased both *IL-1β* (*** *p* < 0.001), *IL-6* (F (3,19) = 245.2, *** *p* < 0.001), *Itgam* (** *p* < 0.01), *AIF1* (F (3,16) = 5.925, * *p* < 0.05), *Adgre1* (*** *p* < 0.001), *CD68* (*** *p* < 0.001) and *NOS2* (F (3,15) = 67.85, *** *p* < 0.001) (see Figure 1A,D–H), whereas the mRNA expression levels of anti-inflammatory IL-10, were unaffected (Figure 1C, *p* > 0.05). At the highest concentration tested (10 μg/mL LPS), *IL-1β* (*** *p* < 0.001), *IL-6* (*** *p* < 0.001), *IL-10* (F (3,15) = 3.827, * *p* < 0.05), *Adgre1* (*** *p* < 0.001), *CD68* (** *p* < 0.01) and *NOS2* (*** *p* < 0.001) were all increased significantly when compared to controls (Figure 1A–C,F–H). No significant changes were observed in the levels of *Itgam* and *AIF1* (Figure 1D,E, *p* > 0.05).

### 2.2. Time-Course Analyses Showing Temporal Gene Expression Changes of Pro-Inflammatory Cytokines and Microglial Activation Markers in LPS-treated BV2 Cells

**Figure 2:**
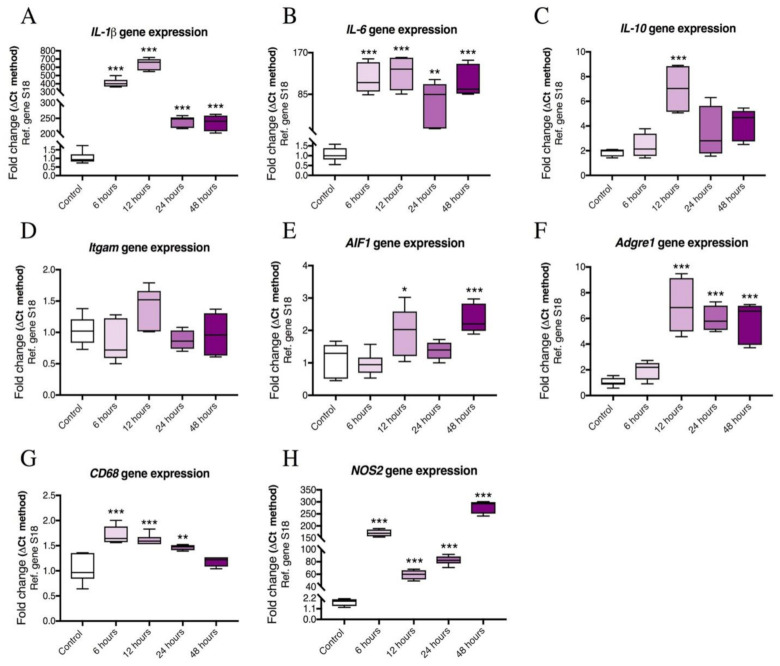
Time-course analyses of the mRNA expression levels of pro-inflammatory markers and cytokines after exposure to LPS. Murine BV2 cells were serum starved for 24 h prior to treatment with 1.0 μg/mL of LPS for 0, 6, 12, 24 and 48 h. The RNA was extracted by TRISURE reagent and reverse transcribed into cDNA. The mRNA levels of (**A**) IL-1β, (**B**) IL-6, (**C**) IL-10, (**D**) Itgam, (**E**) AIF1, (**F**) Adgre1, (**G**) CD68 and (**H**) NOS2 were determined by RT-qPCR with S18 as an internal housekeeping gene. Levels were quantified by Delta-CT method. Data represent means of 4—6 samples for each group. Results are expressed as mean ± SEM. * *p* < 0.05, ** *p* < 0.01, *** *p* < 0.001 vs. control as determined by one-way ANOVA followed by Dunnett’s post hoc test. (*Gene*: protein acronyms): *IL-1β*: Interleukin 1β, *IL-6*: Interleukin 6, *IL-10*: Interleukin 10, *Itgam*: CD11b, *AIF1*: Iba1, *Adgre1*: F4/80, *CD68*: Cluster of differentiation 68 (CD68), *NOS2*: iNOS, *S18*: ribosomal protein S18.

Once it had been established that 1 μg/mL LPS was the most effective concentration to trigger inflammation in our cell model, we sought to determine the temporal response of BV2 cells to treatment. We conducted a time-course analysis of the gene expression profiles of the markers used in the main experiments at five different time points (0, 6, 12, 24 and 48 h). We found that after 6 h exposure to LPS, there were significant increases in *IL-1β* (F (4,25) = 196, *** *p* < 0.001), *IL-6* (F (4,24) = 16.87, *** *p* < 0.001), *CD68* (F (4,23) = 16.99, *** *p* < 0.001) and *NOS2* transcripts (F (4,19) = 262.6, *** *p* < 0.001) (Figure 2A,B,G,H). However, *IL-10*, *Itgam*, *AIF1* and *Adgre1* expression levels did not show significant changes (Figure 2C–F), (*p* > 0.05 vs. control).

After 12 h of LPS exposure, the majority of the pro-inflammatory markers showed significant increases. These included: *IL-1β* (*** *p* < 0.001), *IL-6* (*** *p* < 0.001), *IL-10* (F (4,19) = 7.316, *** *p* < 0.001), *Adgre1* (F (4,25) = 27.15, *** *p* < 0.001), *AIF1* (F (4,25) = 8.547, * *p* < 0.05), *CD68* (*** *p* < 0.001) and *NOS2* (*** *p* < 0.001) (Figure 2A–C,E–H).

BV2 cells exposed to LPS for 24 h resulted in significant increases in *IL-1β* (*** *p* < 0.001), *IL-6* (** *p* < 0.01), *Adgre1* (*** *p* < 0.001), *CD68* (** *p* < 0.01) and *NOS2* (*** *p* < 0.001) when compared with control (Figure 2A–B,F–H).

After 48 h of LPS exposure, *IL-1β* (*** *p* < 0.001), *IL-6* (*** *p* < 0.001), *AIF1* (*** *p* < 0.001), *Adgre1* (*** *p* < 0.001) and *NOS2* genes (*** *p* < 0.001) were all significantly upregulated (Figure 2A,B,E,H). *IL-10*, *Itgam* and *CD68* mRNA levels did not differ from controls at this time point (*p* > 0.05) (Figure 2C,D,G).

### 2.3. Expression of PACAP, VIP and Receptor Transcripts in BV2 Cells Exposed to LPS

**Figure 3:**
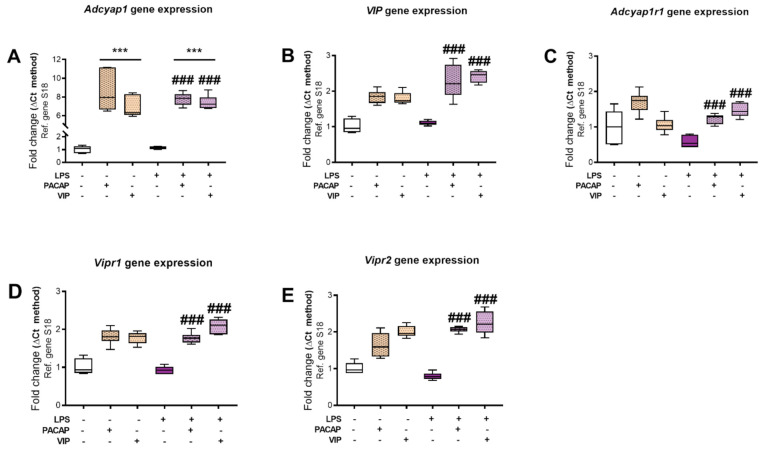
PACAP, VIP and receptors gene expression after treatment with LPS in the presence (or not) of exogenous PACAP or VIP. Real-time qPCR analyses of (**A**) *Adcyap1*, (**B**) *Vip*, (**C**) *Adcyap1r1*, (**D**) *Vipr1* and (**E**) *Vipr2* mRNA levels in BV2 cells that were either left untreated or treated with 100 nM PACAP, 100 nM VIP, 1 μg/mL LPS alone or in the presence of PACAP or VIP after 24 h. Relative changes in mRNA levels were determined using the Delta-CT method and normalized to the ribosomal protein subunit S18, here used as the housekeeping gene. Data represent the mean of 3–6 biological replicates for each group. Results are expressed as mean ± SEM. *** *p* < 0.001 vs. control, ### *p* < 0.001 vs. LPS only as determined by one-way ANOVA followed by Tukey post hoc test. (*Gene*: protein acronyms): *Adcyap1*: PACAP, *Vip*: VIP, *Adcyap1r1*: PAC1 receptor, *Vipr1*: VPAC1 receptor, *Vipr2*: VPAC2 receptor, *S18*: ribosomal protein S18.

In order to evaluate whether LPS perturbs mRNA expression levels of PACAP, VIP and/or their receptors, BV2 cells treated with LPS at 1 μg/mL for 24 h and controls were supplemented with either 100 nM PACAP, or 100 nM VIP [30,31,53,54,55]. In the absence of LPS, stimulation with PACAP (100 nM) or VIP (100 nM) significantly increased PACAP gene (*Adcyap1*) mRNA expression by either 6- or 8-fold (F (5, 29) = 57.91, *** *p* < 0.001) (Figure 3A) but did not reliably increase the expression of the genes encoding for *Vip*, PAC1 receptor (*Adcyap1r1*), VPAC1 receptor (*Vipr1*) or VPAC2 receptor (*Vipr2*) (*p* > 0.05, Figure 3B–E). LPS administration did not affect any of the genes encoding PACAP, VIP or their related receptors (Figure 3A–E); however, co-treatment of LPS-treated cells with PACAP or VIP resulted in a global and significant increase in the transcript levels of both the peptides and their receptors (### *p* < 0.001 vs. LPS, Figure 3A–E).

### 2.4. PACAP or VIP Treatment Rescue LPS-Induced AIF1/Iba1 and NOS2/iNOS Levels in BV2 Microglial Cells

**Figure 4:**
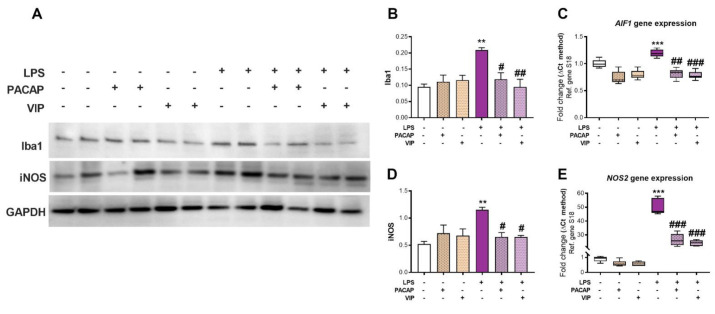
Effects of treatment with PACAP or VIP on LPS-induced microglia activation markers in BV2 cells. Real-time qPCR and Western blot analyses depicting *AIF1*/Iba1 and *NOS2*/iNOS mRNA and protein expression levels in cells exposed to LPS (1 μg/mL) and after co-treatment with either PACAP or VIP at 24 h. mRNA levels of *AIF1* (**C**) and *NOS2* (**E**) and related proteins (Iba1 and iNOS (**A**,**B**,**D**)) are shown. qPCR data represents means of *n* = 5–6 biological replicates for each group. Results are expressed as mean ± SEM. ** *p* < 0.01 or *** *p* < 0.001 vs. control; # *p* < 0.05, ## *p* < 0.01 or ### *p* < 0.001 vs. LPS only, as determined by one-way ANOVA followed by the Tukey post hoc test. Western blot data represent the mean of four independent experiments, each run in duplicate. *AIF1*: Ionized calcium binding adaptor molecule 1 (Iba1), GAPDH: Glyceraldehyde3-phosphate deyhydrogenase, S18: ribosomal protein subunit.

To assess if PACAP and VIP exerted anti-inflammatory effects in BV2 cells, we exposed cell cultures to LPS and measured gene and protein expression of the microglial activation marker *AIF1*/Iba1 (Figure 4A–E). In addition, since production of NO is of particular importance in the pathophysiology of several CNS disorders, and due to the toxicity of its by-products, e.g., peroxynitrates, and because of its known role as an inflammatory mediator [56,57,58], we also assessed the expression levels of *NOS2*/iNOS. We examined the effects of PACAP and VIP in both the presence and absence of LPS stimulation. Our results show that the LPS challenge caused a significant increase in both *AIF1* (F (5, 30) = 24.89, *** *p* < 0.001 vs. control, Figure 4C) and *NOS2* mRNA levels (F (5, 26) = 240.8, *** *p* < 0.001, Figure 4E), accompanied by similar increases in protein expression (Iba1: F (5, 18) = 6.352, ** *p* < 0.01 and iNOS: F (5, 18) = 5.666, ** *p* < 0.01, Figure 4A,B,D). Treatment with either PACAP or VIP inhibited the LPS-induction of both *AIF1* (## *p* < 0.01 and ### *p* < 0.001 vs. LPS, respectively) and *NOS2* gene expression (### *p* < 0.001 vs. LPS for both peptides), as well as the expression of the translated Iba1 (# *p* < 0.05 or ## *p* < 0.01 vs. LPS, respectively) and iNOS proteins (# *p* < 0.05 vs. LPS for both peptides) (Figure 4A,B,D).

To complement our biochemical analyses, we performed immunocytochemistry. BV2 microglia were grown on coverslips under each of the experimental conditions (untreated control, LPS, LPS + PACAP and LPS + VIP) and stained using an antibody to Iba1 (Supplementary Figure S1A,B). Iba1 expression was increased in LPS-only treated cells (F(3, 20) = 28.59, ** *p* < 0.01 vs. Ctrl, Supplementary Figure S1B). After application of either PACAP or VIP, the intensity of the staining was a significantly decreased when compared to the LPS group (### *p* < 0.001 for both PACAP and VIP, Supplementary Figure S1A,B).

### 2.5. PACAP or VIP Treatment Reduce the Expression of pro-Inflammatory Cytokines in BV2 Cells Exposed to LPS

**Figure 5:**
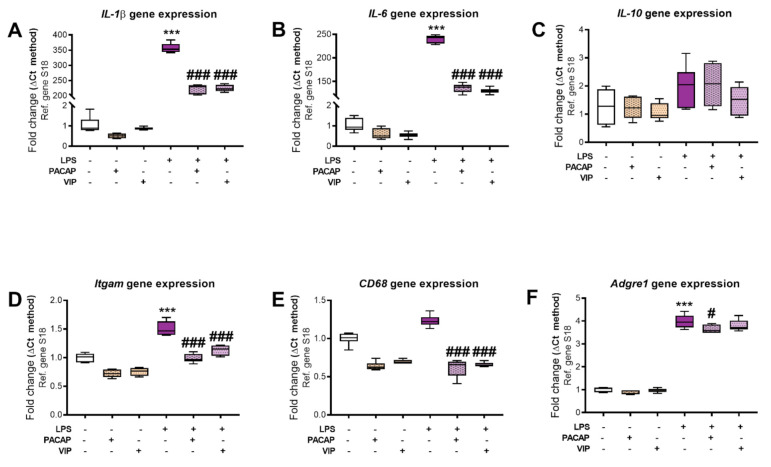
Transcript levels of inflammatory markers after treatment of BV2 cells with LPS alone or in combination with PACAP or VIP. Murine BV2 cells exposed (or not) to LPS (1 μg/mL) for 24 h were co-treated with either PACAP or VIP, and mRNA levels were assessed by real-time qPCR. Expression of *IL-1β* (**A**), *IL-6* (**B**), *IL-10* (**C**), *Itgam* (**D**), *Adgre1* (**E**) and *CD68* (**F**) are shown. Transcript levels were calculated using the Delta-CT method. Data represent the means of 5–6 samples for each group. Results are expressed as mean ± SEM. *** *p* < 0.001 vs. control, # *p* < 0.05 and ### *p* < 0.001 vs. LPS only, as determined by one-way ANOVA followed by Tukey post hoc test. *IL-1β*: Interleukin 1-beta, *IL-6*: Interleukin 6, *IL-10*: Interleukin 10, *Itgam*: CD11b, *Adgre1*: F4/80, *CD68*: Cluster of differentiation 68, S18: ribosomal protein subunit S18.

In these experiments, we evaluated whether PACAP or VIP reduced the heightened levels of pro-inflammatory cytokines in BV2 microglial cells following exposure to LPS. Cells were exposed to LPS and treated as described above. Additional drug-treatment controls were also included. As shown in Figure 5A–F, LPS treatment strongly increased the mRNA expression of *IL-1β* (F5, 30 = 1411, *** *p* < 0.001), *IL-6* (F5, 30 = 1893, *** *p* < 0.001), *Itgam* (F5, 30 = 66.69, *** *p* < 0.001), *Adgre1* (F5, 30 = 443, *** *p* < 0.001) and *CD68* (F5, 30 = 61.94, *** *p* < 0.001) compared to untreated groups. Of note, the mRNA expression levels of the anti-inflammatory cytokine *IL-10* remained unchanged (Figure 5C, *p* > 0.05).

PACAP treatment of cells exposed to LPS significantly decreased the mRNA expression levels of *IL-1β* (### *p* < 0.001), *IL-6* (### *p* < 0.001), *Itgam* (### *p* < 0.001), *Adgre1* (# *p* < 0.05) and *CD68* (### *p* < 0.001) when compared with the LPS-only group (Figure 5A,B,D–F). PACAP treatment did not affect *IL-10* mRNA expression levels (Figure 5C, *p* > 0.05).

VIP treatment of cells exposed to LPS produced similar patterns of change to those seen following PACAP treatment. Specifically, there were significant reductions in *IL-1β* (### *p* < 0.001), *IL-6* (### *p* < 0.001), *Itgam* (### *p* < 0.001) and *CD68* mRNAs (### *p* < 0.001) (Figure 5A,B,D,F). Once again, VIP did not affect *IL-10* gene expression (Figure 5C, *p* > 0.05). *Adgre1* transcript levels were also reduced, although this decrease was not statistically significant (Figure 5F, *p* > 0.05).

### 2.6. PACAP or VIP Treatment Reduce the Release of Nitrites in the Culture Media

**Figure 6:**
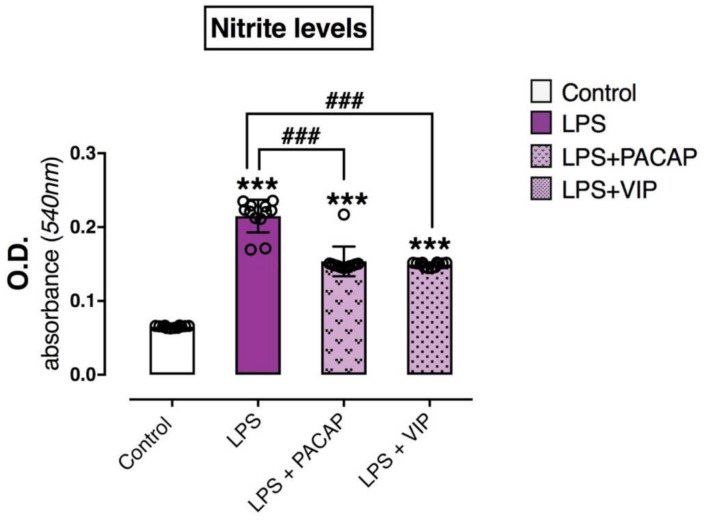
Release of nitrates in BV2 cells exposed to LPS and following treatment with PACAP or VIP. BV2 cells plated at 5 × 10^5^ in 25 cm^2^ flasks were starved with 1% full growth media (FGM) for 24 h prior to treatment with 10% FGM (control), 1.0 μg/mL LPS, 1.0 μg/mL LPS + 100 nM PACAP and 1.0 μg/mL LPS + 100 nM VIP for 24 h. Media were then replaced with treatment-free media and allowed a further 24 h incubation to allow NO accumulation. The culture medium was then aspirated and subjected to Griess reaction to obtain the readings using an ELISA reader (540 nm filter). This experiment was repeated with at least two independent batches of BV2 cells in two separate experiments. Results are expressed as mean ± SEM. *** *p* < 0.001 vs. control, ### *p* < 0.001 vs. LPS-treated cells, as determined by one-way ANOVA followed by Tukey post hoc test. O.D: Optical density.

To determine whether the downregulation of *NOS2*/iNOS induced by PACAP or VIP was paralleled by reductions in the level of nitrites secreted by the cells, the relative abundance of NO was measured in the culture media using the Griess method [59]. As shown in Figure 6, the LPS-treated group showed a substantial and significant increase in nitrite levels when compared with the control (F3, 44 = 201.4, *** *p* < 0.001) (Figure 6). Treatment with PACAP or VIP significantly reduced LPS-evoked NO release (### *p* < 0.001 for PACAP and VIP, respectively), although it did not return to control levels (*** *p* < 0.001 for PACAP and VIP, respectively).

### 2.7. Distinct Effects of PACAP or VIP Treatment on LPS-Stimulated BV2 Cell Morphology

When BV2 cells were challenged with LPS and co-treated with either PACAP or VIP, we observed gross morphological differences in the cells between the two treatments. To systematically describe these differences, we conducted morphometric analyses of untreated cells, or cells exposed for 24 h to either LPS alone or in combination with PACAP or VIP.

**Figure 7:**
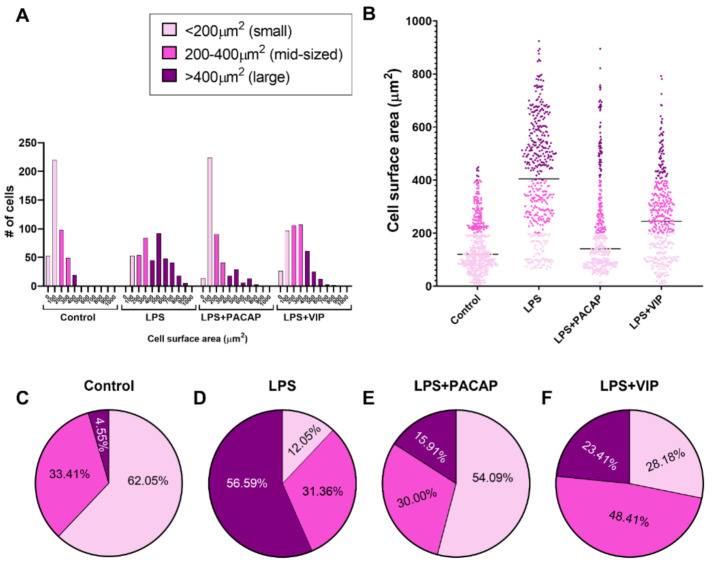
Redistribution of BV2 cell subpopulations based on cell surface area after treatment with LPS or in combination with PACAP or VIP. BV2 cells plated at 5 × 10^5^ in 25 cm^2^ flasks were starved with 1% FGM for 24 h prior to treatment with 10% FGM (control), 1.0 μg/mL LPS, 1.0 μg/mL LPS + PACAP 100 nM and 1.0 μg/mL LPS + VIP 100 nM for 24 h; then, images were captured using Nikon Eclipse Ts2 inverted microscope. Images were assigned to investigators who were blind to experimental conditions and cells were classified as either small (<200 μm^2^), mid-sized (200–400 μm^2^) or large (> 400 μm^2^) (please refer to Table 1 for further details on cells characteristics). Analyses were performed using Image J 1.51 (NIH) software. Measurements of cell surface area were performed on a total of 1760 cells (440 cells × treatment group). (**A**) Distribution of cell populations based on cell surface area identify distinct patterns across treatments. (**B**) Scatter plot of cell surface area distribution in each treatment group (the black line depicts the median). (**C**–**F**) Pie charts showing the percentage of distribution of cell populations after ranking into small, mid-sized or large cells.

**Figure 8:**
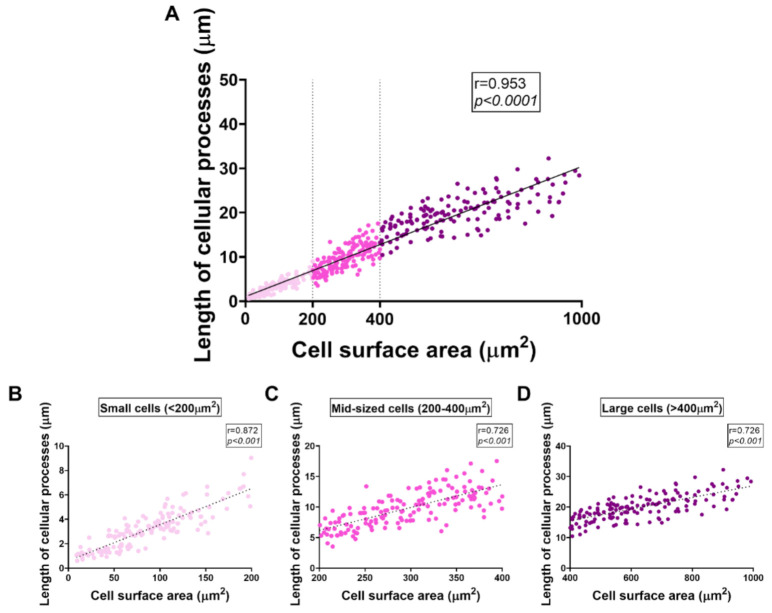
Correlation between cell surface area and length of cellular processes. BV2 cells plated at 5 × 10^5^ in 25 cm^2^ flasks were starved with 1% FGM for 24 h prior to treatment with 10% FGM (control), 1.0 μg/mL LPS, 1.0 μg/mL LPS + PACAP 100 nM and 1.0 μg/mL LPS + VIP 100 nM for 24 h; then, images were captured using Nikon Eclipse Ts2 inverted microscope. Images were assigned to investigators who were blind to experimental conditions. Measurements of cellular processes were performed using Image J 1.51 (NIH) software. The average length of cellular processes (expressed in μm) was obtained by averaging the measurements of 2–3 processes × cell. (**A**) Correlation between cell surface area and the length of cellular processes. Regression analyses showing significant correlations between cell surface area and length of cellular processes both in (**B**) small cells (<200 μm^2^), (**C**) mid-sized cells (200–400 μm^2^) and (**D**) large cells (>400 μm^2^).

Measurements of cell surface areas in the BV2 microglial cell lineage revealed a broad heterogeneity among cells, with somata surface areas ranging from very small to very large (min size = 7 μm^2^, max size = 924 μm^2^). This large heterogeneity of cell sizes across treatment groups prompted us to conduct a distribution analysis aimed at determining if each treatment resulted in a distinct pattern of cell distribution as a function of their size. As shown in Figure 7A,B, the ranking of cells based on surface area revealed three distinct cell subgroups; (i) small cells = < 200 μm^2^; (ii) mid-sized cells = 200–400 μm^2^; and (iii) large cells = > 400 μm^2^) whose patterns of changes were distinctly affected across our treatment groups. We detected a strong correlation between cell surface area and the length of cellular processes (Pearson’s *r* = 0.953, **** *p* < 0.0001, Figure 8A), which was maintained across each of the three cell size categories identified (*r* = 0.872 for small cells, *r* = 0.726 for mid-sized cells and *r* = 0.726, *** *p* < 0.001, respectively, Figure 8B–D). On the basis of this analysis, we used these features to classify BV2 cell sizes in subsequent morphological experiments.

In cells exposed to LPS, the number and percentage of cells presenting with a large soma was strongly increased when compared with untreated controls (249/440 (56.69%) vs. 20/440 (4.55%) in controls, Figure 7C,D). As predicted, these results were associated with a drastic reduction in the number of small-sized cells (53/440 (12.05%) vs. 273/440 (62.05%) in controls), but no changes in the number of mid-sized cells (138/440 (31.36%) vs. 147/440 (33.34%), Figure 7C,D).

When LPS-exposed BV2 cells were co-treated with PACAP, we observed a major shift towards a phenotype characterized by small BV2 cells (238/440 (54.09%) vs. 53/440 (12.05%), Figure 7A,B), and a robust reduction in the number of large-sized cells (70/440 (15.91%) vs. 249/440 (56.69%) was observed in LPS-treated cells, Figure 7D,E). Similar to untreated or LPS-treated cells, PACAP treatment did not affect the number of mid-sized cells (132/440 (30.00%) vs. 147/440 (33.34%).

BV2 cells that were exposed to LPS and co-treated with VIP showed a distinct redistribution of cells, based on their cell surface areas (Figure 7A,B). In contrast to PACAP-treated cells, VIP treatment triggered only a partial shift towards small-sized cells (124/440 (28.18%) vs. 238/440 (54.09%) in PACAP-treated cells, Figure 7D,F). In contrast, we observed a predominant shift towards mid-sized cells (213/440 (48.41%), Figure 7F), an effect not seen in any of the other treatment groups (138/440 (31.36%) in controls, 147/440 (33.34%) in LPS-treated cells and 132/440 (30.00%) in PACAP-treated cells, respectively, Figure 7C–F).

### 2.8. VIP but Not PACAP Treatment Causes the Phenotypic Shift of BV2 Cells towards Mid-Sized Spindle/Bipolar-Shaped Cells after LPS Challenge

Once we had established that PACAP and VIP each differed in their abilities to segregate cell subpopulations within a distinct cell size-based category, we sought to determine whether changes in cell size might also relate to changes in cell morphology. Therefore, stereological analyses were undertaken in BV2 cells, treated as indicated above. In physiological conditions, BV2 cells exhibit three distinct morphologies: rounded, bipolar/spindle and multipolar shapes.

**Figure 9:**
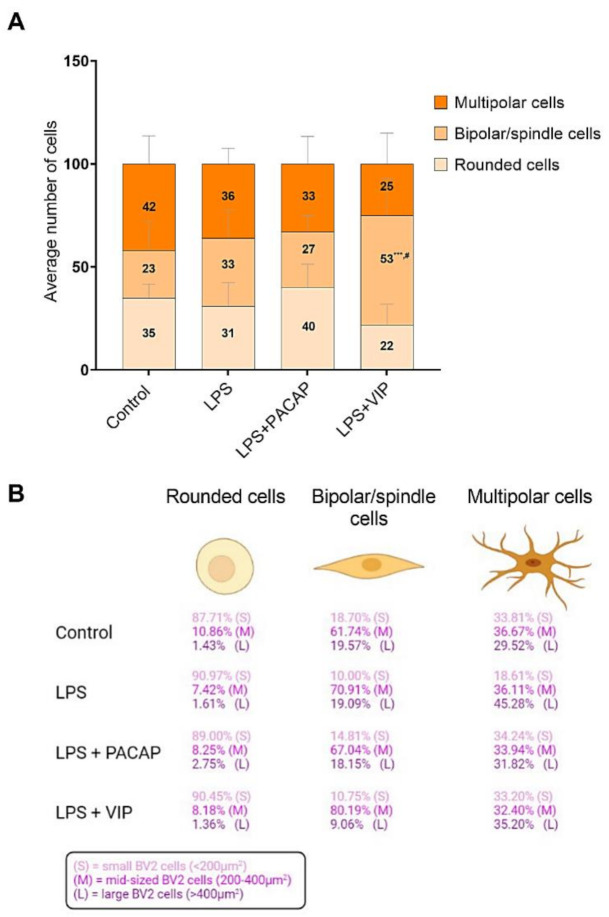
Redistribution of BV2 microglial cell subpopulations based on morphology after treatment with LPS only, LPS + PACAP and LPS+VIP. BV2 cells plated at 5 × 10^5^ in 25 cm^2^ flasks were starved with 1% FGM for 24 h prior to treatment with 10% FGM (control), LPS (1.0 μg/mL), LPS + PACAP (100 nM) and LPS + VIP (100 nM) for 24 h. Images were captured using Nikon Eclipse Ts2 inverted microscope. (**A**) Average number of cells presenting with either rounded, bipolar/spindle or multipolar shape in each experimental condition. (**B**) Percentage of BV2 cells for each morphology that exhibited either a small (S), mid-sized (M) or large soma (L). Investigators conducting stereological and morphometric measurements were blind to the experimental conditions. Measurements and cell counts were performed using Image J 1.51 (NIH) software. An average of 100 cells per experimental condition were analysed, with each condition replicated using ten different batches of cells (*n* = 10). *** *p* < 0.001 vs. control or # *p* < 0.05 vs. LPS-treated cells, as determined by two-way RM ANOVA followed by Tukey post hoc test.

In comparing cell subpopulations, two-way RM ANOVA and a Tukey post hoc test revealed ligand-selective effects of VIP and PACAP treatment × cell morphology interaction (** *p* < 0.01). Specifically, within the LPS+VIP group, we found that cells exhibiting a bipolar/spindle shape were the most frequently represented cell population (53%), both in comparison with control groups (23%, *** *p* < 0.001 vs. control) or LPS-treated cells (33%, # *p* < 0.05 vs. LPS) (Figure 9A).

None of the other cell morphologies (i.e., rounded or multipolar) were significantly different across treatment groups (*p* > 0.05) (Figure 9A).

In-depth analyses revealed that within each phenotype (i.e., rounded, spindle or multipolar cells), treatment with either LPS alone or in combination with PACAP or VIP triggered a redistribution of the percentage of cells of a given size category (i.e., small, mid-sized and large cells). Specifically, we found that about 90% of all rounded BV2 cells were small sized (>200 µm^2^), irrespective of treatment. Conversely, analyses of bipolar/spindle-shaped cells showed that between 60–70% of these cells were mid-sized. However, in VIP-stimulated cells, this percentage increased to 80.19% (Figure 9B), suggesting that VIP treatment not only affects the total population of bipolar-shaped cells, but also promotes their clustering into mid-sized cells in a unique manner.

Analyses of multipolar cells demonstrated that under the experimental conditions tested, this phenotype was not specifically associated with any cell size category with a varying percentage of 33–36% of multipolar cells falling either into a small, mid-sized or large size category (Figure 9B). The only exception was seen in the LPS group, where almost half of all the multipolar cells had a large soma (45.28%), followed by 36.11% mid-sized and only 18.61% of small-sized cells. Additional morphological details are shown in Table 1.

### 2.9. PACAP and VIP Differentially affect BV2 Cell Motility and Urokinase Plasminogen Activator (uPA) Expression after LPS Challenge

Microglia are highly motile cells that constantly monitor their microenvironment; they migrate quickly to sites of injury where they phagocytose debris and dying cells [60]. To determine if the distinct morphological changes observed after PACAP or VIP treatment were associated with functional changes, a scratch/wound healing assay was performed in BV2 cells exposed to LPS, in the presence, or not, of PACAP or VIP.

**Figure 10:**
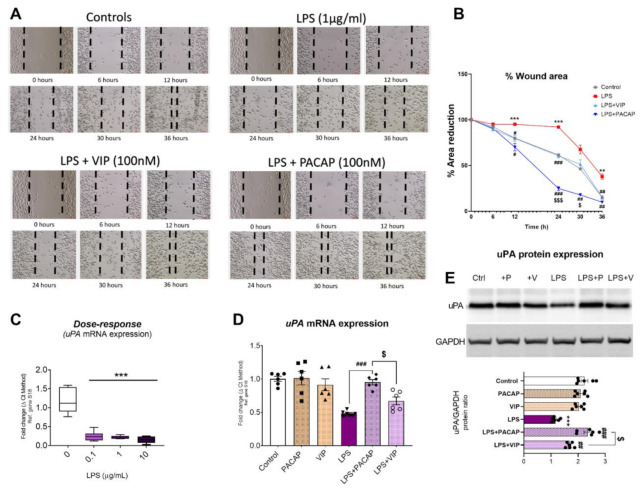
Effects of PACAP or VIP treatment on cell motility (wound healing assay) and uPA expression in LPS-treated BV2 cells. BV2 cells plated in 6-well plates and left to grow until 95% confluent in 1% FGM for 24 h prior to treatment with 10% FGM (control). A scratch was created in the centre of the well using a P1000 pipette tip and media replaced with indicated treatments: 10% FGM (control), 1.0 μg/mL LPS, 1.0 μg/mL LPS + PACAP 100 nM and 1.0 μg/mL LPS + VIP 100 nM. (**A**) Images were taken over time at 0, 6, 12, 25, 30 and 36 h using Nikon Eclipse Ts2 inverted microscope. Black dashed lines delineate the wound area. Magnification = 20×. Scale bar = 60 μm. (**B**) Analyses were performed using Image J 1.51 (NIH) software, where the area reduction was calculated. ** *p* < 0.01 or *** *p* < 0.001 vs. time control at the corresponding time point; # *p* < 0.05, ## *p* < 0.01 or ### *p* < 0.001 vs. LPS at the corresponding time point; $ *p* < 0.05 or $$$ *p* < 0.001 vs. LPS + VIP. (**C**) Real-time qPCR analyses showing the effects of increasing concentrations of LPS (0, 0.1, 1 and 10 μg/mL) on uPA mRNA expression. (**D**,**E**) Real-time qPCR and Western blot analyses depicting *uPA* mRNA and protein expression levels in BV2 cells exposed to LPS and treated with either PACAP or VIP at 24 h. mRNA and protein levels of uPA are shown. Real-time qPCR data represent means of *n* = 5–6 biological replicates for each group, whereas Western blot data represent the mean of four independent experiments. Results are expressed as mean ± SEM. *** *p* < 0.001 vs. control; # *p* < 0.05, ## *p* < 0.01 or ### *p* < 0.001 vs. LPS only; $ *p* < 0.05 vs. LPS+VIP, as determined by one-way ANOVA followed by Tukey post hoc test. uPA: urokinase plasminogen activator, GAPDH: Glyceraldehyde3-phosphate deyhydrogenase, S18: ribosomal protein subunit.

LPS treatment almost completely abrogated cell motility in BV2 cells during the initial 24h, resulting in a significantly increased residual wound area in LPS vs. control cells (*** *p* < 0.001 both at 12 and 24 h). Cell motility was partially recovered at 30 h (*p* > 0.05 vs. control); however, the percentage of the wound area was still significantly larger than in controls at 36 h (*** *p* < 0.001 vs. control at 36 h, Figure 10A,B).

VIP treatment effectively reduced LPS-induced immobility, recording a significant reduction in the wound area at 12 h (# *p* < 0.05 vs. LPS), 24 h (### *p* < 0.001) and 36 h (## *p* < 0.01) (Figure 10A,B).

Similar to VIP, treatment with PACAP also significantly increased the percentage of wound area closure starting from 12 h (# *p* < 0.05 vs. LPS), with further increases recorded at later time points (### *p* < 0.001 vs. LPS at 24 h, ## *p* < 0.01 at 30 h and ## *p* < 0.01 at 36 h, respectively, Figure 10A,B). At 24 and 30 h, the percentage of PACAP-induced wound closure was significantly higher than in VIP treated cells ($$$ *p* < 0.001 vs. LPS + VIP at 24 h and $ *p* < 0.05 vs. LPS + VIP at 30 h, respectively) (Figure 10A,B).

To provide the molecular basis to explain the different activities of PACAP and VIP on cell motility, we analysed the expression of urokinase-type plasminogen activator (uPA), a plasminogen-cleaving enzyme known to be involved in cell migration [61]. Dose–response studies with increasing concentrations of LPS (0.1–10 μg/mL) demonstrated that the inflammatory mimetic significantly down-regulated *uPA* mRNA expression at all doses tested (*** *p* < 0.001) (Figure 10C). PACAP treatment in LPS-treated cells significantly increased *uPA* mRNA (### *p* < 0.001 vs. LPS, Figure 10D) and protein expression (### *p* < 0.001, Figure 10e), whereas VIP treatment failed to rescue LPS-induced downregulation of *uPA* mRNAs (*p* > 0.05 vs. LPS, Figure 10D) but partly rescued uPA protein expression (## *p* < 0.01 vs. LPS, Figure 10E). Of note, the different activities of PACAP and VIP on *uPA* mRNA and protein were both statistically significant ($ *p* < 0.05 vs. LPS + VIP for both, Figure 10D,E).

## 3. Discussion

In the present study, we demonstrate that both PACAP and VIP exert potent immunosuppressive activities in BV2 microglial cells exposed to lipopolysaccharide (LPS), an inflammatory mimetic. By exposing healthy BV2 murine microglia cells to LPS, we attempt to replicate in vitro the functions of resident microglial cell populations in response to stimulation by PAMPs or DAMPs. We show that the peptides downregulated the expression of a range of pro-inflammatory mediators following LPS induction whilst also altering gene expression for PACAP/VIP and their receptors. Additionally, morphological and morphometric evaluations of this cell line revealed patterns of phenotypic redistributions of cell subpopulations that were distinct for cells treated with either peptide during an LPS challenge. We report for the first time that PACAP- or VIP-induced BV2 phenotypes are characterized by distinct effects on cell motility and the expression of uPA, an enzyme involved in regulating this biological function. When taken together, the data from this study provide a better understanding of the mechanisms through which these naturally occurring peptides may dampen inflammation or affect cell surveillance functions by microglia.

Investigating the effects that LPS and peptide addition have on the PACAP/VIP family is pivotal to uncover individual peptide functions. In the literature, studies of PACAP-deficient mice have demonstrated that PACAP plays critical roles in the endogenous cytoprotective machinery. In these knockout phenotypes, mice show increased vulnerability to stressors in the nervous system, and they exhibit distinct morphological and behavioural abnormalities [62,63,64]. In our microglia cell model, the striking increases observed in *Adcyap1* expression in cells treated with VIP was an unexpected finding and given the well-known neuroprotective effect of the encoded peptide, we suggest a strong translational potential [65,66,67].

Despite their homology, VIP and PACAP genes have different promoters. PAC1 receptors that are able to couple to different signalling pathways are expressed in these cells, it would be expected, therefore, that VIP and PACAP are able to control different biological activities. VIP is able to prevent activated microglia-induced neurodegeneration in models of brain trauma in mice, confirming its immune-modulatory role in vivo [48]. We surveyed levels of *Vip* mRNAs in response to immune challenge with LPS. The changes showed a marked recovery of expression following LPS + PACAP/VIP treatments in comparison to both resting and LPS only (Figure 3A,B).

PAC1, VPAC1 and VPAC2 levels are diminished after LPS treatment and are partially restored by exogenous peptide supplementation. *Adcyap1r1*, also known as PAC1 receptor, has a higher affinity to PACAP over VIP. Studies in PAC1 receptor knockout (KO) mice demonstrate that endogenous PACAP and the activation of PAC1 receptor exerts anti-inflammatory effects, as the mice exhibited higher levels of *IL-6* [68,69]. Similarly, PACAP and VIP are more protective against the lethal effects of systemic LPS administration in wild-type mice than in PAC1 receptor KO mice through the activation of the receptor [68,69]. Our observations at the mRNA level show increased *Adcyapr1r1* gene expression after treatment with LPS + PACAP/VIP in comparison with LPS only. *Vipr1* and *Vipr2*, also known as VPAC1 and VPAC2 receptors, respectively, have equally high affinity for both PACAP and VIP peptides. Earlier studies show that by using a VPAC1 receptor agonist on LPS-treated cells, it reduced *TNF* levels and *IL-6* in vitro, and also that VIP treatment was able to inhibit pro-inflammatory cytokine production from monocytes, mainly through the VPAC1 receptor [70]. VPAC2 KO mice exhibit exacerbated clinical, histopathological and immunological features of experimental autoimmune encephalomyelitis (EAE) in comparison to wild-type (WT) mice [71]. In our studies, *Vipr1* gene expression is upregulated post-LPS + PACAP/VIP treatment, and levels are increased in comparison with control and LPS only (Figure 3D). Likewise, *Vipr2* gene expression is upregulated post treatment (Figure 3E). Nonetheless, in both animal and human models the selective activation of VPAC1 receptors has been shown to be more efficacious in controlling immune responses in comparison to VPAC2 [72], which may explain the trend that we observed within our study. It is reasonable, then, for us to hypothesize that the changes in the PACAP/VIPergic system in BV2 cells result in a neuroprotective and immunomodulating influence on microglia, as there is increased activation of the receptors following LPS treatment and exogenous administration of PACAP and VIP. Furthermore, we confirm that both of the receptors are expressed, and at least one of these is functional in BV2 microglia cells. Further we show that receptor activity is inversely correlated with the extent of the immune insult, as when there is immune insult, there is decreased receptor expression.

The imbalance between the excessive pro-inflammatory and suppressed anti-inflammatory cascade processes is responsible for the consequent neurodegeneration and damage to microglia cells. Resting microglial cells lack or express only low levels of the inducible nitric oxide synthase (iNOS), the main enzyme capable of synthesizing nitric oxide (NO), so we anticipated that we would not observe high expression of the gene in the control group [73]. The amounts of basal iNOS and NO observed in the control groups are likely attributable to constitutive activity in BV2 cells in response to the immortalisation process, which is obtained through the introduction of a retrovirus in the cell lineage [74,75]. Increased levels of *NOS2* have been associated with brain inflammation and neurodegenerative diseases as the mechanism that activated microglia intoxicate neurons in culture has been suggested to involve the increased release of NO [56,76,77]. A reduction in neuronal cell death has been shown in studies utilizing iNOS inhibitors and knockout mice, resulting in protection of the animal to the immune challenge, conveying the neurotoxic effect of iNOS/*NOS2* [78,79]. This is due to the fact that the iNOS promoter contains discreet regions where one is a NF-κB binding site, activated mainly by LPS [80,81]. In our studies, we saw decreased *NOS2* expression in activated BV2 cells post-LPS + PACAP/VIP treatment, affirming the anti-inflammatory capabilities of both PACAP and VIP (Figure 4). This result was also confirmed through protein analyses. It is known that NO is released from microglia following exposure to LPS [82], a finding further supported in our experiments, where the BV2 cells accumulated nitrite as a stable oxidized product of NO within the culture media when stimulated with LPS, and PACAP/VIP were able to inhibit this production (Figure 6). These observations confirmed our hypotheses, which were based on observations from previous studies that showed that PACAP/VIP had a modulatory effect on iNOS through a reduction in NF-κB binding [83,84].

The release of inflammatory mediators, such as *IL-1β*, *IL-6* and *Itgam*, defines the activation state of the cells. These inflammatory cytokines allow for the initiation of inflammatory cascade, and thus result in damage. In the present study, the levels of pro-inflammatory cytokines, including *IL-1β*, *IL-6*, *Itgam*, *Adgre1* and *CD68* were significantly increased with LPS, and these levels were restored following treatments with the peptides (Figure 5).

Another crucial step in the microglial injury response is the upregulation of the specific microglia marker Iba1. *AIF1* is the gene encoding Iba1, a binding peptide associated with microglial activation in the brain [85]. The specificity of Iba1 as a specific microglia marker is convincingly demonstrated by a study showing that while cultured hypothalamic neurons do not stain positively for Iba1, a massive 94% of cells in a primary culture of microglia were positively stained [86]. Iba1 expression in microglia is also shown to increase in models of brain disease, whilst it remains low prior to disease presentation [35]. In addition, Iba1 mRNAs and proteins are detectable in cultured BV2 cells and expression is reliably increased after LPS stimulation [50,87]. Similar to iNOS, our findings showed that Iba1 is also expressed at low levels by untreated control cells (Figure 4), which could again be due to the immortalised nature of the BV2 cell lineage, which may induce some spontaneous polarization of cells. Nonetheless, we present compelling experimental data that show the upregulation of *AIF1*/Iba1 after exposure to LPS and a clear downregulation of its expression following treatment with PACAP or VIP (Figure 4). These findings are seen at both mRNA (Figure 4C) and protein levels (Figure 4A,B) and are also corroborated by immunocytochemical evidence (Figure 4F,G). This shows that, at least in vitro, PACAP and VIP modulate microglial activation. This observation could lead to potential options to dampen neurodegeneration. We also conclude that Iba1 is an early marker of microglial activation when accompanied by increases in *IL-6* and *IL-1β*, as we observed coincident changes within 12 h of the onset of inflammation. These results are noteworthy given that both BV2 cells, and primary microglia express Iba1, enabling translation of PACAP and VIP abilities to primary microglia [82].

Transformation of cellular morphology is another crucial characteristic alerting to the presence of activation-induced changes in microglia [88,89]. Such morphometric assessments may also aid in the identification of microglial functional state. We investigated changes in morphology, along with Iba1 staining in an attempt to unveil morphological/morphometric patterns that could suggest changes in BV2 microglial activation, or perhaps other biological functions. Nevertheless, it is important to note that this is a novel morphometric approach and at present, not much supporting literature is available.

Morphometric analyses demonstrated that BV2 cells exhibited a range of sizes, with the cell surface areas ranging from as little as 7 μm^2^ up to 924 μm^2^. This prompted us to assess whether the distribution of cell subpopulations varied across the treatment groups with distinctive and specific patterns. In our analyses, we distinguished three clear subsets/categories of cell sizes (small cells = <200 μm^2^, mid-sized cells = 200–400 μm^2^ and large cells = >400 μm^2^), whose pattern of changes were affected by LPS exposure and, importantly, that were found to be affected by PACAP or VIP treatment (Figure 7). Using linear regression analyses, we also determined that cell size correlated with the length of cellular processes (Figure 8A), and that this relationship was maintained across each of the cell size categories (Figure 8B–D), supporting the suitability of this ranking method to classify BV2 cells according to size.

Untreated cells were predominantly small-sized (62.05%, Figure 7C). LPS caused a major shift towards the population of large-sized cells (56.59%, Figure 7D) at the expenses of small cells (reduced to 12.05% by LPS), suggesting a correlation between cell size and polarization state. PACAP treatment of LPS-stimulated microglia rescued the small cell subpopulation (54.09%, Figure 7E) and reduced the portion of large-sized cells (reduced from 15.91%), supporting a possible correlation between microglial functional state (i.e., resting or activated) and soma size. Of note, the subpopulation of mid-sized BV2 cells did not vary significantly among untreated, LPS-treated or *LPS + PACAP*-treated cells (33.41%, 31.36% and 30%, respectively, Figure 7C–E). In contrast, VIP treatment only partly rescued the percentage of small-sized cells (28.18%) but caused a robust increase in the proportion of mid-sized cells (48.41%, Figure 7F).

Gross evaluation of BV2 cell morphology revealed a heterogeneous appearance of cells, which, presented with three distinct phenotypes (i.e., rounded-, spindle/bipolar- or multipolar-shaped cells), associated with peculiar changes in morphological features (please refer to Table 1). There was a uniform distribution of rounded, bipolar or multipolar cells across treatments (Figure 9A). Yet, similarly to morphometric results, only cells in the LPS+VIP group displayed a significant increase in the subset of cells exhibiting bipolar/spindle morphology (53%, Figure 9A), suggesting a possible relationship between the latter and cell size (mid-sized cells) under these experimental conditions.

To explore the possible link between BV2 cell morphology and size, cells were selected in function of their morphological appearance; cell surface areas were measured and ranked accordingly across treatment groups (Figure 9B). Most of the rounded cells (about 90%), irrespective of treatment, were categorized as small-sized. Similarly, about 60–70% of cells with a bipolar shape were mid-sized; however, in the LPS+VIP group the percentage of mid-sized cells increased to about 80% (Figure 9B). Based on these findings, we suggest that BV2 microglia (and perhaps even primary microglia) are subject to a whole spectrum of morphological/morphometric changes that may be accompanied by increased or decreased cell functionality [90,91].

Microglial cells are involved in many biological processes, including immune surveillance, phagocytosis, as well as tissue repair and cell migration [14,92,93]. Therefore, we assessed if the distinct morphological and morphometric changes seen after VIP, but not PACAP, treatment could be associated with distinct biological effects of either peptide. We focused our attention on cell mobility, as this function is associated strongly with changes in cell shape [94]. Additionally, previous studies have reported that PACAP reliably stimulates the expression and activity of the urokinase plasminogen activator, an enzyme involved in microglial mobility [95], both in peripheral glial cells [96] and the prefrontal cortex and hippocampus of mice [97]. To our surprise, evaluation of cell motility, measured using the wound healing/scratch assay, revealed that PACAP was more effective than VIP in reinstating cell mobility after an LPS challenge (Figure 10A,B). Furthermore, PACAP completely rescued the LPS-induced reduction in uPA expression, whereas VIP was only partly effective (Figure 10D,E). Based on these findings, it cannot be excluded that mid-sized bipolar-shaped BV2 cell subpopulations may be less motile than their counterparts and/or contribute to hindering the overall microglial motility, although this warrants further investigations. We also identified a mechanism by which PACAP, more effectively than VIP, stimulates uPA expression to restore cell mobility, suggesting that uPA itself may be able to affect BV2 cell morphology, size and, consequently, motility. However, additional investigations are needed to confirm this theory.

In conclusion, our investigations have demonstrated that both PACAP and VIP are two potent immune modulatory peptides that should be considered further as potential targets for the treatment of neurodegenerative conditions, especially when an inflammatory component is present. In BV2 microglia, PACAP or VIP stimulation during an LPS challenge reliably reduced the expression of pro-inflammatory cytokines, microglial activation markers and NO release. Furthermore, treatment with these peptides triggered distinct changes in the morphology and size of cells, which were in parallel with distinct abilities of PACAP or VIP to stimulate cell migration/motility.

Our findings corroborate the idea that PACAP or VIP can exert distinctive regulatory activities on certain aspects of microglial function and reveal different implications for the use of one ligand over the other for therapeutic purposes. Such differences are likely to be explained by the different binding activities of PACAP and VIP to their receptors.

## 4. Materials and Methods

### 4.1. Cell Culture

The study was carried out in murine microglial cells, BV2 kindly provided by Dr Eryn Werry from the University of Sydney, Sydney, Australia. Cells were cultured in Dulbecco’s modified Eagle’s medium (DMEM), supplemented with either 1% or 10% heat-inactivated foetal bovine serum (FBS) and 1% penicillin (100 IU/mL)/streptomycin (100 μg/mL) (Sigma-Aldrich, St. Louis, MO, USA). Cells were incubated at 37 °C in a humidified atmosphere with 5% CO_2_. After seeding at a density of 4 × 10^5^ cells for 24 h, BV2 cells were serum starved for a further 24 h in media containing 1% FBS prior to treatments.

Cells were subjected to different treatments: (1) control, (2) 100 nM PACAP (Sigma-Aldrich, St. Louis, MO, USA), (3) 100 nM VIP (AusPep, Tullamarine, Australia), (4) 1 μg/mL LPS (Sigma-Aldrich, St. Louis, MO, USA), (5) LPS + PACAP and (6) LPS + VIP. Thereafter, cells were harvested at 6, 12, 24 or 48 h for downstream molecular analyses.

### 4.2. Quantitative Real Time Polymerase Chain Reaction

Total RNA extracts from harvested cells were subjected under certain treatments (*n* = 3–6) and isolated using 1mL TRIsureTM reagent (Bioline, Sydney, Australia), 0.2 mL chloroform and precipitated with 0.5 mL isopropanol (Sigma-Aldrich, St. Louis, MO, USA). The pellet was washed with 75% ethanol and air dried by inversion of tubes. Total RNA (1 μg) was diluted in milliQ H_2_O to a final volume of 11 μL. A total of 9 μL of Tetro cDNA synthesis mix (Bioline, Sydney, Australia) was added to each 11 μL aliquot, each containing: 1 μL oligo (dT)18 primers, 1 μL random hexamer primers mix, 1 μL 10 mM dNTP mix, 4 μL 5× RT buffer, 1 μL RNase inhibitor and 1 μL reverse transcriptase. A volume of 20 μL of each sample were then incubated at 25 °C for 10 min, followed by 45 °C for 30 min. The reaction was terminated by incubation of samples at 85 °C for 5 min.

Aliquots of cDNA were amplified using specific primers shown in Table 2. Hard-Shell^®^ 96-Well PCR plates (Bio-Rad, Hercules, CA, USA) were utilised, and up to 4 genes were tested per run. Each PCR reaction mix was prepared in a final volume of 7 μL, to which we added 3 μL of cDNA (final concentration = 30 ng) and conducted using the CFX96 Touch™ Real-Time PCR Detection System (Bio-Rad, Hercules, CA, USA). Each PCR reaction mix contained 5 μL SensiFAST SYBR No-ROX kit (Bioline, Sydney, Australia), 0.8 μL 5 μM forward primer, 0.8 μL 5 μM reverse primer and 0.4 μL milliQ H_2_O. PCR was performed using the following settings:

(1) 95 °C for 2 min, (2) 60 °C for 10 s, (3) 72 °C for 10 s, (4) plate read, (5) repeat step 2 for 45 cycles, (6) 65 °C for 35 s, (7) plate read and (8) repeat step 6 × 60 times using the PCR instrument. After the protocol was completed, the Ct values were exported and analysed. Relative changes in gene expression were computed using the comparative Ct method [51]. The formula 2^−ΔΔCt^ was used to calculate fold changes. Baseline measurements for untreated controls were set to 1.

### 4.3. Western Blot Analysis

Protein lysate was homogenised in RIPA buffer (Sigma-Aldrich, St. Louis, MO, USA), which was supplemented with cOmplete™ULTRA protease inhibitor cocktail (Roche Life Science, North Ryde, NSW, Australia). Lysates were then sonicated twice at 50% power for 10 s using an ultrasonic probe, followed by centrifugation prior to protein quantification. Protein concentrations were determined using the Bicinchoninic Acid (BCA) Assay Kit (ThermoFisher Scientific, Waltham, MA, USA).

Protein samples (15 μg) were separated by sodium dodecyl sulphate (SDS)-polyacrylamide gel electrophoresis (SDS-PAGE) using 4–20% Mini-PROTEAN^®^ TGX Stain-Free™ protein gels (15 wells) (Bio-Rad, Hercules, CA, USA). Samples were prepared by 3.75 μL of 4× Laemmli buffer (Bio-Rad, Hercules, CA, USA) and β-mercaptoethanol (Sigma-Aldrich, St. Louis, MO, USA) mix (Laemmli buffer 20:1 with β-mercaptoethanol) to a final volume of 15 μL. Samples were then denatured at 70 °C for 10 min using the T100™ thermal cycler.

Proteins (15 μg) were resolved on gels and transferred to a PVDF membrane using the semi-dry approach (Trans-Blot Turbo™ Transfer Pack; Bio-Rad, Hercules, CA, USA). Once proteins were transferred, the membrane was removed from the apparatus and briefly washed in tris-buffered saline containing Tween^®^20 (TBST) 1× three times before blocking with 5% blocking buffer containing 5% skim milk in TBST 1× for 1 h at room temperature at 55–60 rpm. The membranes were probed with anti-rabbit primary antibodies recognizing either Iba1, iNOS or GAPDH (used as loading control) overnight at 4 °C under gentle oscillation (55 rpm) (for a full list of the antibodies used, please refer to Table 3). Following incubation, membranes were washed thoroughly. Finally, membranes were incubated with sondary antibodies for 1 h at room temperature and washed again before imaging. To visualize immunoreactive bands, we used Clarity™ Western ECL Blotting Substrate (Bio-Rad, Hercules, CA, USA). Images were acquired on the ChemiDoc™ MP System (Bio-Rad, Hercules, CA, USA). Band intensities were quantified using ImageJ software and values were normalized to GAPDH.

### 4.4. Immunocytochemistry

BV2 microglial cells were grown on cover slips pre-coated in poly-l-lysine (Sigma-Aldrich, St. Louis, MO, USA) and fixed with 4% paraformaldehyde (PFA) (Sigma-Aldrich, St. Louis, MO, USA). Then, cells were permeabilised in PBS containing 0.25% Triton X-100 (Sigma-Aldrich, St. Louis, MO, USA) for 10 min and then rinsed with PBS. Thereafter, fixed cells were treated with blocking solution (PBS, 1% goat serum albumin (Sigma-Aldrich, St. Louis, MO, USA) and 0.1% Tween^®^20) for 30 min. Blocking serum was replaced with appropriately diluted primary antibody (in PBS) (polyclonal anti-rabbit Iba1 antibody; GeneTex, Irvine, CA, USA) and incubated at 4 °C overnight with gentle agitation. Cells were rinsed with PBS for 3 changes for 5 min each. A volume of 1 mL of appropriately diluted fluorescein-conjugated sondary antibody (in PBS) (anti-rabbit IgG FAb2 Alexa Fluor^®^ 488 molecular probes; Cell Signalling Techology, Danvers, MA, USA) was applied to the wells and incubated at room temperature for 1 h protected from light. Following the sondary antibody incubation, cells were washed with PBS. A total of 0.3 μg/mL DAPI stain (Cell Signalling Techology, Danvers, MA, USA) was applied to the cells for 1 min and discarded. Cells were washed again with PBS and then coverslips were mounted onto one-frosted ended microscope slides (Australian Scientific, NSW, Australia) using mounting medium (ProLong^®^ Gold Antifade Reagent; Cell Signalling Techology, Danvers, MA, USA). Clear nail polish was applied to the edges of the coverslips to ensure that they remain sure and sealed. Colours of antibody staining were observed with the Delta Vision Elite deconvolution microscope. Relative fluorescence was determined using the open-source software ImageJ. Nuclei were defined using the DAPI channel (435–485 nm emission) using a 420 nm longpass filter, whereas Iba1 immunosignal was captured using the Fluorescein Iso-thiocyanate (FITC) channel and collected through a 525/50 nm bandpass filter. To avoid the possibility of DAPI spectral bleed-through into Alexa fluor 488 dye, fluorescent signals were captured and recorded on separate channels using sequential imaging and merged afterwards. Regions of Interest (ROIs) used to quantify the integrated intensity of fluorescent images were selected by applying a macro with a preset threshold level and gamma, brightness and contrast values, which were kept constant throughout the experiments. Background values were extrapolated from at least three separate fixed areas taken from the same photomicrograph. To calculate relative fluorescence intensity, we applied the following formula:

*Relative fluorescence = Integrated density of stained area − (mean background area × mean fluorescence of background readings*)

### 4.5. Nitrite Assay

Levels of nitric oxide (NO) in culture supernatants were quantified by the utilisation of the Griess reaction. Briefly, BV2 cells (5 × 10^5^) were seeded in 25 cm^2^ flasks and starved in 1% FGM for 24 h, prior to initiating the six treatments for 12 h. Following treatments, culture media was replaced with freshly prepared media not containing any treatment and cells were incubated for a further 24 h to allow accumulation of NO. Twenty-four hours later, the supernatant was aspirated and collected. Griess reagent (Sigma-Aldrich, St. Louis, MO, USA) was prepared by dissolving 1 g Griess reagent in 25 mL milliQ H_2_O (1:25 w/vol) in an autoclaved bottle shielded from the light. Under sterile conditions, 100 μL of conditioned media was added into a 96-well plate, using at least 6 replicates from each group. Each experiment was repeated at least twice. An amount of 100 μL of Griess reagent was added to each well containing 100 ul of conditioned media from each group. The plate was incubated at room temperature for 15 min, protected from light. Absorbance using a 540 nm filter was measured by the Tecan infinite M1000 Pro ELISA reader which reflected nitrite levels. Changes in NO were normalized to that of untreated cells and are expressed as percentage of controls.

### 4.6. Morphological Analyses

BV2 cells (5 × 10^5^) were seeded in 25 cm^2^ flasks and starved in 1% FGM for 24 h, prior to initiating treatments: control, LPS only, LPS + 100 nM PACAP and LPS + 100 nM VIP for 24 h. Images were then captured using a Nikon Eclipse TS2 inverted microscope and subjected to morphological assortment and analyses (magnification 20×). Four experimenters blind to the cell culture conditions were assigned random images to analyse. After measuring the average diameter and length of cellular processes, along with soma surface area (average measurements from 450 individual cells) and assessing the gross morphology (i.e., flattened cell body, multipolar/bipolar/rounded shape), cells were then grouped into three different categories: (a) small, (b) mid-sized, and (c) large cells (please see Table 1). Analyses of microscope images were performed using ImageJ 1.51 (NIH, Bethesda, MD, USA; available at http://rsb.info.nih.gov.ezproxy.lib.uts.edu.au/ij/, first accessed in 19 June 2018), where cells were labelled and measured individually.

### 4.7. Wound Healing Assay

BV2 cells were seeded onto 6-well plates at a density of 1 × 10^5^ cells/mL and left until they were 90–95% confluent. Scratches were created using a P1000 pipette tip to scratch a straight line on the culture plate, reference lines on the bottom of the plate were used for alignment and to obtain the same field for each image. PBS was used to remove detached cells. Fresh media with indicated treatments was added: Control, LPS only, LPS + 100 nM PACAP and LPS + 100 nM VIP. Images were then captured at 0, 6, 12, 24, 30 and 36 h using a Nikon Eclipse TS2 inverted microscope. The scratch area was determined and used to calculate the % area reduction over time using ImageJ. For further details on image analyses, please refer to Supplementary File S2.

### 4.8. Statistical Analysis

Data were analysed using GraphPad Prism^®^ version 7.02 software (GraphPad Software Inc., La Jolla, CA, USA). All data were tested for normality with the Kolmogorov–Smirnov test. All variables were normally distributed. Datasets are reported as mean ± standard error of mean (SEM). One-way or two-way repeated measures (RM) analysis of variance (ANOVA) followed by post hoc tests (as appropriate) were utilised to assess which groups were statically different. Result for all statistical tests were considered significant when *p* ≤ 0.05.

## Figures and Tables

**Table 1:** Morphological features of BV2 microglial cells.

Category	Description	Process Diameter (μm)	Process Length (μm) ± SD	Surface Area (µm^2^)	Visual Example
Small	Mostly round or oval shaped cells (87–91%), with small soma and thin, short/absent processes	˂2 or none	(min = 0, max = 9.3) mean 3.17 ± 1.56	<200	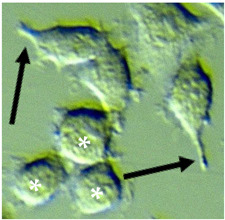
Mid-sized	Predominantly spindle-shaped cells (70–80%) with flat appearance, variable length and mainly thick processes	2–4	(min = 3.54, max = 17.54) mean 9.79 ± 2.88	200–400	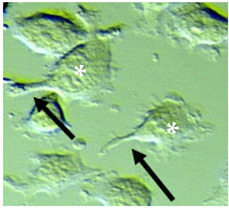
Large	Mixed multipolar (30-45%) or spindle-shape appearance (9–19%), flattened cells with hypertrophied soma, with thick, elongated processes	2–4	(min = 10.45, max = 32.24) mean 19.90 ± 4.09	>400	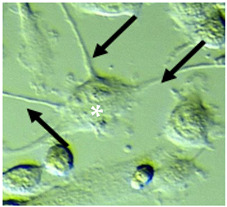

Criteria for assortment of BV2 cells into small, mid-sized and large cells. Descriptions are listed with an approximate visual example. Black arrows indicate cellular processes. White asterisks depict different morphologies of cells.

**Table 2:** Primer sequences used for RT-qPCR.

Gene	Forward	Reverse	Bp Length
*Adcyap1* Acc# NM_009625.2	CTGCGTGACGCTTACGCCCT	CCTAGGTTCTCCCCCGCGCC	152
*Vip* Acc# NM_011702.2	TGGCAAACGAATCAGCAGCAGCA	AGCCATTTGCTTTCTGAGGCGGG	106
*Adcyap1r1* Acc# NM_007407.3	CAGTCCCCAGACATGGGAGGCA	AGCGGGCCAGCCGTAGAGTA	139
*Vipr1* Acc# NM_011703.4	TCAATGGCGAGGTGCAGGCAG	TGTGTGCTGCACGAGACGCC	127
*Vipr2* Acc# NM_009511.2	GCGTCGGTGGTGCTGACCTG	ACACCGCTGCAGGCTCTCTGAT	155
*IL-1β* Acc# NM_008361.4	GCTACCTGTGTCTTTCCCGT	CATCTCGGAGCCTGTAGTGC	164
*IL-6* Acc# NM_031168.2	CCCCAATTTCCAATGCTCTCC	CGCACTAGGTTTGCCGAGTA	141
*IL-10* Acc# NM_010548.2	GCATGGCCCAGAAATCAAGG	GAGAAATCGATGACAGCGCC	91
*Itgam* Acc# NM_001082960.1	GAGCAGGGGTCATTCGCTAC	GCTGGCTTAGATGCGATGGT	94
*Adgre1* Acc# NM_001355722.1	GCTTATGCCACCTGCACTGA	GGTGAGTCACTTTGAAGACATTCG	143
*AIF1* Acc# NM_001361501.1	GCTTTTGGACTGCTGAAGGC	GCTTCAAGTTTGGACGGCAG	114
*CD68* Acc# NM_001291058.1	CTCCCACCACAAATGGCACT	CTTGGACCTTGGACTAGGCG	95
*NOS2* Acc# NM_010927.4	AATCTTGGAGCGAGTTGTGG	CAGGAAGTAGGTGAGGGCTTG	139
*uPA* Acc# NM_008873.3	CATCCATCCAGTCCTTGCGT	TTTCATGGTAGTGCCGCTGG	87
*S18* Acc# NM_011296.2	CCCTGAGAAGTTCCAGCACA	GGTGAGGTCGATGTCTGCTT	145

Forward and reverse primers were chosen from the 5′ and 3′ region of each gene messenger ribonucleic acid (mRNA). The right column displays the expected length of each PCR amplification product.

**Table 3:** Antibodies used in Western Blots.

Antibody	Source	Predicted Band Size	Dilution
PLAU	LS-C193095	55 kDa	1:1000
Iba1	GTX100042, GeneTex	17 kDa	1:300
iNOS	GTX60599, GeneTex	32 kDa	1:1000
GAPDH	VPA00187, Bio-Rad	37 kDa	1:1500
Goat anti Rabbit IgG HRP (Secondary)	STAR208P, Bio-Rad		1:20,000

Antibodies used for Western Blots and their predicted molecular weights are shown. All primary antibodies are raised in rabbits and are polyclonal. The dilutions used are depicted in the right column.

## Supplementary Materials

The following are available online at https://www.mdpi.com/article/10.3390/ijms222010947/s1.
