# Supplementary material for: PACAP and VIP Modulate LPS-Induced Microglial Activation and Trigger Distinct Phenotypic Changes in Murine BV2 Microglial Cells
Source: Int J Mol Sci. 2021 Oct 11;22(20):10947. doi: 10.3390/ijms222010947 (PMC8535941; doi:10.3390/ijms222010947)
Supplement: Supplementary file 1 [file ijms-22-10947-s001.zip › Supplementary Figure S1.pdf]

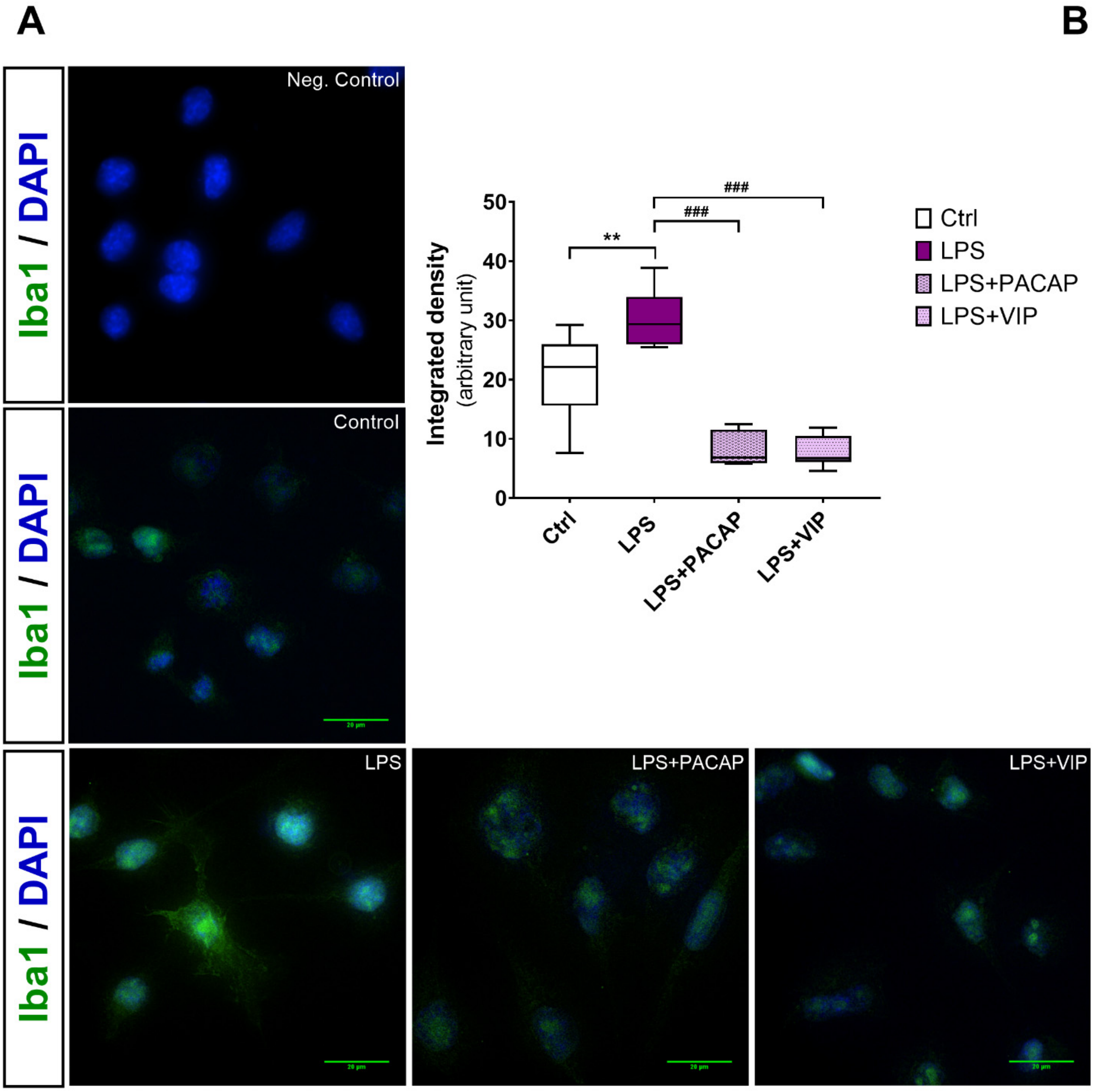

Supplementary Figure S1. ***Iba1-like immunoreactivity in BV2 cells after treatment with LPS and cotreatment with PACAP or VIP.*** Representative immunocytofluorescence photomicrographs (**A**) and bar graph showing semi-quantification of Iba1 fluorescent intensity (**B**) in BV2 cells exposed to LPS (1µg/ml) and co-treated with either PACAP or VIP for 24h. Nuclei were counterstained with DAPI. Results are expressed as mean ± SEM. * p<0.05 vs. control, ### p<0.0001 vs. LPS-treated cells, as determined by one-way ANOVA followed by Sidak's post-hoc test. At least six images were taken from each treatment group from two separate experiments where one representative image is shown. Primary antibody was omitted in the negative control. Magnification = 63.5×. Scale bar = 20µm.
