# Supplementary material for: PACAP and VIP Modulate LPS-Induced Microglial Activation and Trigger Distinct Phenotypic Changes in Murine BV2 Microglial Cells
Source: Int J Mol Sci. 2021 Oct 11;22(20):10947. doi: 10.3390/ijms222010947 (PMC8535941; doi:10.3390/ijms222010947)
Supplement: Supplementary file 1 [file ijms-22-10947-s001.zip › Supplementary File S2.pdf]

Methodology used to measure wound area using ImageJ

1. Open image
2. Image → Type → 8-bit
3. Image → Adjust → Threshold → reset
4. Process → FFT → Bandpass Filter → OK
5. Image → Adjust → Threshold
6. Process → Filters → Miminum → Radius 7 pixels → OK
7. Magic wand → click wound area (the selected wound area is contoured in yellow)
8. Analyze → Measure

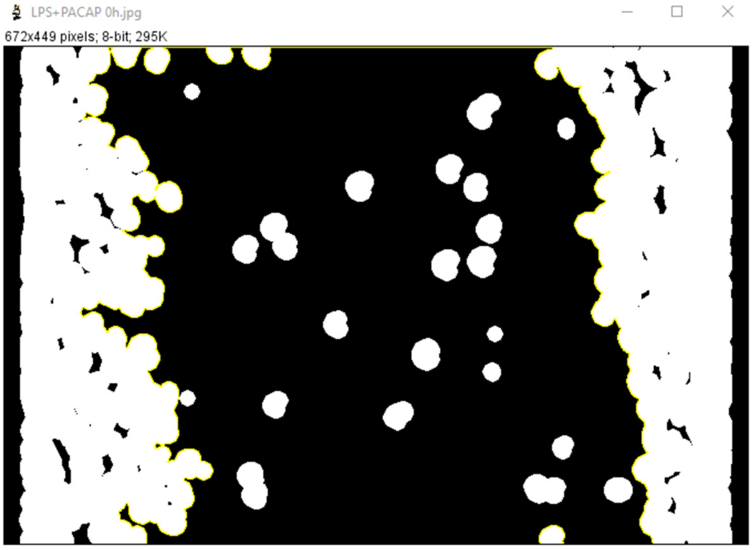

9. Image → Adjust → Threshold → unclicked Dark background
10. Analyse → Tools → ROI manager
11. Manually select each cell (within the wound area) using the Wand Tool and clicked Add (or press "t" (in this example 24 cells were found to occupy the wounded area)

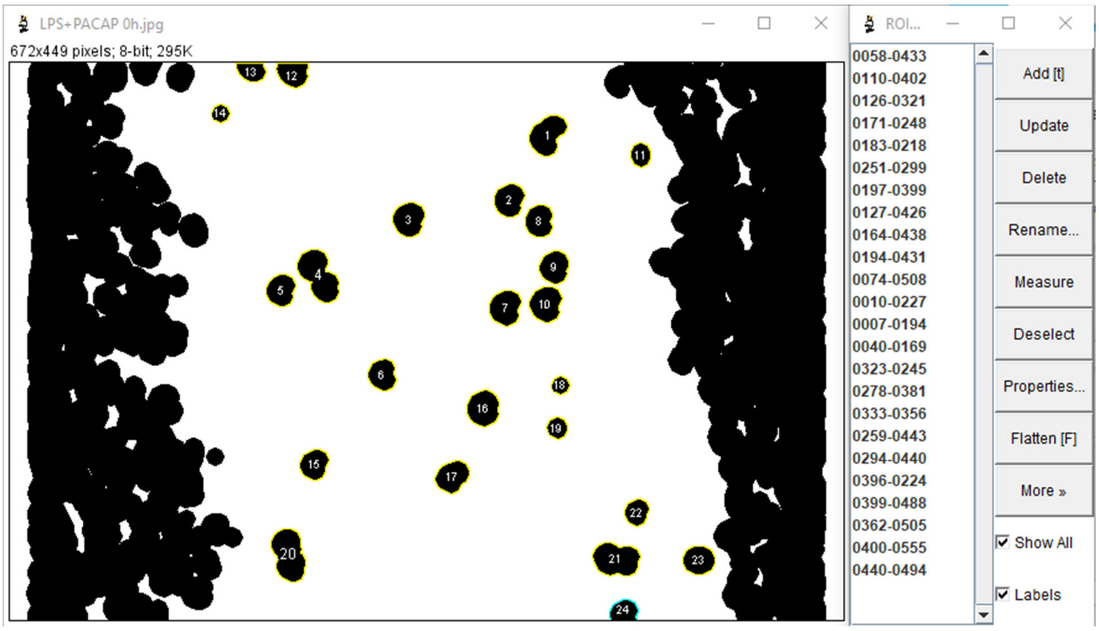

12. Analyze → Measure

Results

File Edit Font Results

	Label	Area	Mean
1	LPS+PACAP 0h.jpg	184093	134.43
2	LPS+PACAP 0h.jpg:0058-0433	689	90.75
3	LPS+PACAP 0h.jpg:0110-0402	514	86.61
4	LPS+PACAP 0h.jpg:0126-0321	563	80.98
5	LPS+PACAP 0h.jpg:0171-0248	922	82.00
6	LPS+PACAP 0h.jpg:0183-0218	479	92.75
7	LPS+PACAP 0h.jpg:0251-0299	456	90.02
8	LPS+PACAP 0h.jpg:0197-0399	578	84.00
9	LPS+PACAP 0h.jpg:0127-0426	474	87.65
10	LPS+PACAP 0h.jpg:0164-0438	499	91.39
11	LPS+PACAP 0h.jpg:0194-0431	591	84.87
12	LPS+PACAP 0h.jpg:0074-0508	251	110.59
13	LPS+PACAP 0h.jpg:0010-0227	417	75.07
14	LPS+PACAP 0h.jpg:0007-0194	294	86.75
15	LPS+PACAP 0h.jpg:0040-0169	161	112.00
16	LPS+PACAP 0h.jpg:0323-0245	443	85.84
17	LPS+PACAP 0h.jpg:0278-0381	606	46.68
18	LPS+PACAP 0h.jpg:0333-0356	529	82.09
19	LPS+PACAP 0h.jpg:0259-0443	161	113.00
20	LPS+PACAP 0h.jpg:0294-0440	226	106.35
21	LPS+PACAP 0h.jpg:0396-0224	889	82.70
22	LPS+PACAP 0h.jpg:0399-0488	816	71.59
23	LPS+PACAP 0h.jpg:0362-0505	329	101.32
24	LPS+PACAP 0h.jpg:0400-0555	484	75.29
25	LPS+PACAP 0h.jpg:0440-0494	323	94.50

13. Copy results in Excel sheet → Subtract the area of each cell (values in row 2-25) from the total wound area (highlighted in black in the table above)
14. Repeat steps 1-13 for all the images x treatment/condition
15. Average results in each condition and normalise as % of control
